# New insights of the application of water or ethanol-water plant extract rich in active compounds in food

**DOI:** 10.3389/fnut.2023.1118761

**Published:** 2023-03-28

**Authors:** Anna Plaskova, Jiri Mlcek

**Affiliations:** Department of Food Analysis and Chemistry, Faculty of Technology, Tomas Bata University in Zlin, Zlin, Czechia

**Keywords:** active compounds, phytochemicals, antioxidants, food applications, plant-based, fortification, natural products, plant extracts

## Abstract

Plants are recognized as natural sources of antioxidants (e.g., polyphenols, flavonoids, vitamins, and other active compounds) that can be extracted by green solvents like water, ethanol, or their binary mixtures. Plant extracts are becoming more used as food additives in various food systems due to their antioxidant abilities. Their application in food increases the shelf life of products by preventing undesirable changes in nutritional and sensory properties, such as the formation off-flavors in lipid-rich food. This review summarizes the most recent literature about water or ethanol-water plant extracts used as flavors, colorings, and preservatives to fortify food and beverages. This study is performed with particular attention to describing the benefits of plant extract-fortified products such as meat, vegetable oils, biscuits, pastries, some beverages, yogurt, cheese, and other dairy products. Antioxidant-rich plant extracts can positively affect food safety by partially or fully replacing synthetic antioxidants, which have lately been linked to safety and health issues such as toxicological and carcinogenic consequences. On the other hand, the limitations and challenges of using the extract in food should be considered, like stability, level of purity, compatibility with matrix, price, sensory aspects like distinct taste, and others. In the future, continuous development and a tendency to use these natural extracts as food ingredients are expected, as indicated by the number of published works in this area, particularly in the past decade.

## 1. Introduction

**Figure 1:**
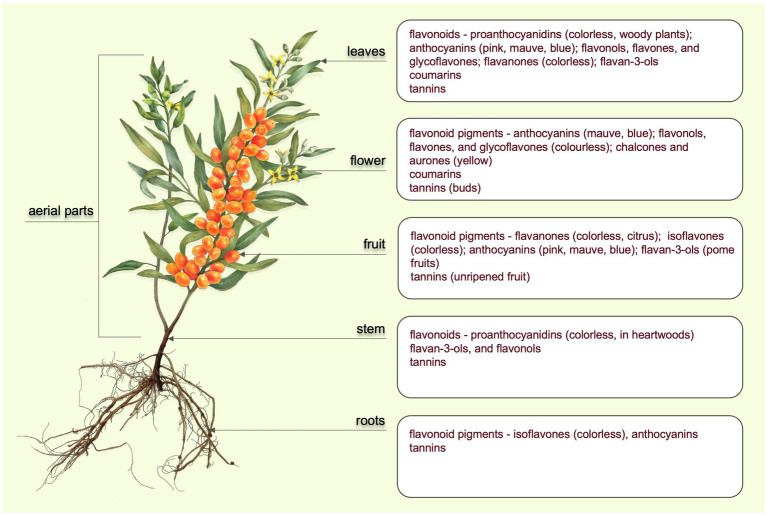
Parts of a plant and the active phenolic compounds found in them (5–7).

Since ancient times, medicinal plants and herbal teas have been used in everyday human life as traditional medicines due to their healing and therapeutic properties (1, 2). Medicinal plants are the source of many phytochemicals which are secondary metabolites responsible for herbs’ beneficial properties (3). Plant secondary metabolism could be bio-synthetic source of several compounds which are attractive to utilize in the food industry and the pharmaceutical, chemical, cosmetic, and agronomic industries. The fundamental aim of plants is to create secondary metabolites as a defense mechanism against microorganisms, insects, and consumers such as herbivores (4). These secondary metabolites include many biologically active substances, for example, alkaloids, essential oils, flavonoids, bitters, coumarins, tannins, glycosides, and saponins. Figure 1 shows the locations of target phenolic compounds in different parts of a plant. Many studies have discussed biologically active compounds as they have antioxidant, antibacterial, antifungal, anti-inflammatory, and anti-tumor effects (8–10). In recent years, plants and their extracts containing high content of bioactive or aromatic compounds have found use in numerous applications in the food industry as flavors, colorings, antioxidants and antimicrobial agents (preservatives), nutrient enhancers, enzymes, and packaging additives (2, 11).

Plants should be appropriately identified, and a health risk assessment should be undertaken before food application (12); for example, the elimination factor for plants could be if they contain heavy metals, mycotoxins, or residues from chemical treatment or fertilization (13, 14). Chemical compounds’ concentration in plants depends on the soil, climate, agricultural practices, irrigation water quality, plant treatment, harvest season, and environmental pollution (10, 13). As a result, to reduce consumer risks, it is important to avoid harvesting plants in areas of high industrial and traffic activities due to the possibility of contamination of heavy metals (14, 15). Moreover, good agricultural practices should be implemented while applying chemical fertilizers or pesticides. Plant material before the extraction process usually requires pre-treatment, which includes washing, drying, enzymatic processes, fermentation, and grinding. Due to the washing treatment, the loading of plant samples with heavy metal contaminants such as aluminum and lead might be slightly reduced (14).

Plant material pre-treatment prevents bioactive compounds’ degradation and the development of undesirable microorganisms, allowing the plant’s shelf life to be extended (16). Drying is one of the most popular plant material preservation methods because water is necessary for microorganisms and plant enzymes to function correctly (16, 17). Dehydration inhibits certain metabolic processes, such as enzymatic degradation, that might break down the bioactive compounds and change sensory and nutritional properties (16–18). Choosing the correct drying treatment is important since it might significantly lose heat-labile molecules, volatile compounds, flavonoid components, and attractive color pigments in plants (16, 19–21). High drying temperatures, on the other hand, may inhibit enzymes in plants that break down the bioactive compounds (16). The freeze-drying technique has the potential to stabilize, enrich, and prolong the life of plants containing bioactive temperature-sensitive molecules without destroying their chemical structure (16). According to a few authors, it is more appropriate to use freeze-drying than air drying at room temperature in the dark or in the microwave to maintain the maximum amount of carotenoids and chlorophylls in herbs (3, 22). However, high drying temperatures might inhibit enzymes in plants that can break down the bioactive ingredients (16).

Dried plants might be milled into a powder before extraction because increasing the contact surface area between the plant matrix and the solvent might increase the extract’s yield and concentration of bioactive molecules through better segregation (23). On the other hand, the fragmentation method used for homogenizing herbs can reduce the yield of volatile molecules such as essential oils since they are released during the technological process, during which the secretory structure is destroyed (24). For example, the concentration of essential oil in peppermint granules (0.8–2.0 mm) was reduced by around 71% compared to the uncrushed plant (24, 25).

Isolation of some bioactive molecules from plant matrices using only solvents during extraction might be challenging due to their bounds with components of cell walls (16, 26). Enzymatic treatment is an effective strategy that might improve the efficiency of extraction target bioactive compounds by better releasing these substances by degrading cell walls and membranes (16, 26). In addition, this pre-treatment offers the possibility of greener chemistry due to the shorter extraction duration, lower solvent usage, and milder conditions (16, 27). Enzyme-assisted extraction can improve phenolic and flavonoid recovery, hence increasing the antioxidant activity of the extracts (28–31). For example, Chew et al. used enzyme pre-treatment of the mulberry leaves with pectinase before ultrasound-assisted water extraction and fortifying cottage cheese with enhanced mulberry extract (28).

A proper extraction method is required to provide a higher yield with a considerable concentration of bioactive components. The various structure of bioactive components might complicate the extraction process because of differences in polarity and solubility (32). Furthermore, the extraction method and various processing parameters strongly influence the extract’s yield, composition, and antioxidant activity; consequently, it is necessary to optimize this process (23, 33). In the last decade, attention has been focused on extraction techniques to limit the use of hazardous substances such as toxic solvents for human health and the environment while minimizing energy requirements and using renewable material as part of a green chemistry strategy (34, 35). Moreover, non-toxic and easy-to-handle solvents for plant extraction are preferred in the food industry (23); for example, water is the safest and least expensive green solvent (36). Water is efficient solvent in extracting polar molecules while in the case of less polar compounds, greater efficiency could be achieved by using organic solvents or binary solvents (36–38). For example, bioactive components such as polyphenols might be poorly soluble in water but have better solubility in organic solvents (39). Water might enhance the efficiency of the extraction process by helping the diffusion of extractable components like polyphenols through plant tissues (40, 41). Furthermore, a low-viscosity solvent may be preferred for preparing plant extract due to the possibility of accelerating mass transfer (42). For example, acidified water or organic solvent (e.g., ethanol or methanol) are effectively used to prepare plant extract rich in anthocyanins (43). Tannins are phenolic compounds with high molecular weights that form colloids when dissolved in water (44, 45). Furthermore, the solubility of tannins is variable, and depending on the target molecules, solvents with different polarities like water, ethanol, methanol, and acetone, as well as their aqueous solutions, might be used for extraction (7, 44, 45). Flavonoid glycosides are more water soluble than aglycones; additionally, both groups may be extracted with binary mixtures of water-alcohol and pure alcohols (46, 47). Furthermore, flavonoids’ hydroxyl groups and sugar moieties influence solubility in water (46). In contrast, less polar flavonoids such as flavanones, flavanols, isoflavones, and methylated flavones might be extracted with ethyl acetate, diethyl ether, chloroform, and dichloromethane (47). Alkaloids are often found in plants as salts of organic acids that are water-soluble under acidic circumstances (48, 49). Moreover, solubility in water increases due to the replacement of organic acid in alkaloid salts by inorganic acid salt (49).

Binary solvent mixtures (e.g., water and organic solvent) are preferred over their neat counterparts because all bioactive molecules in plant material cannot be extracted using only a single solvent (36). In addition, binary solvent like aqueous acetone is better for the extraction of flavanols with higher molecular weight (50). A proper concentration of aqueous and organic solvents could efficiently extract active compounds (50); for example, ethanol is miscible in water, and the relative polarity of binary ethanol-water solvents (80:20, cl/L) is 0.710, which is closer to the value for methanol than ethanol (38). Additionally, the mixture of water and alcohol (ethanol) could be more successful solvent mixture than single solvent in the extraction of polyphenols (41, 51). An organic solvent like methanol might effectively extract moderately polar and polar phenolic molecules (e.g., flavonoid glycosides and phenolic acids), especially those with lower molecular weight (42, 52). This hazardous alcohol might be a concern for food safety. The maximum limit for methanol residues in extracted food components and food is 10 mg/kg; however, the methanol residues should be below 1.5 mg per kg of foodstuff with applied extracts from aromatic plants used as flavorings (23, 53).

Solvents used in producing plant extracts might be removed at the end of the processing step (e.g., by evaporation on a rotary evaporator); however, some residual solvents might remain unintentionally (54). Their level is limited due to quality and safety concerns (53, 55); moreover, their maximum limitations are shown in Table 1 below.

**Table 1:** Properties of solvents arranged according to decreasing polarity (38, 53, 55, 56).

Solvent	Formula	Solubility in H_2_O	Relative polarity^a^ most polar ↓least polar	Max. residue limits in food (preparation of flavorings, extraction solvents)	Residual solvents in drugs^b^
		(g/100 g)	(−)	(mg/kg)	(−)
Water	H_2_O	-	1.000	-	
Methanol	CH_4_O	Miscible	0.762	1.50	Class 2
Ethanol	C_2_H_6_O	Miscible	0.654		Class 3
Propan-1-ol	C_3_H_8_O	Miscible	0.648	1.00	Class 3
Butan-1-ol	C_4_H_10_O	7.7	0.586	1.00	Class 3
Propan-2-ol	C_3_H_8_O	Miscible	0.546	1.00	Class 3
Butan-2-ol	C_4_H_10_O	18.1	0.506	1.00	Class 3
Acetone	C_3_H_6_O	Miscible	0.355		Class 3
Ethylmethylketone	C_4_H_8_O	25.6	0.327	1.00	Class 3
Dichloromethane	CH₂Cl₂	1.32	0.309	0.02	Class 2
Methyl acetate	C_3_H_6_O_2_	24.4	0.253	1	Class 3
Ethyl acetate	C_4_H_8_O_2_	8.7	0.228		Class 3
Hexane	C_6_H_14_	0.0014	0.009	1.00	Class 2
Cyclohexane	C_6_H_12_	0.005	0.006	1.00	Class 2

a A range of normalized values between 0 and 1.000.

b Solvents of Class 2 have less severe toxicity, and solvent residues should be limited to protect human health. Class 3 of solvents are associated with low toxic potential, which good manufacturing procedures or other quality-based standards should limit (55).

The technology extraction factors influencing the composition of water or ethanol-water plant extracts include the solvent (ethanol concentration, mineral composition of water), the extraction time and temperature, the plant-to-solvent ratio, the size and part of plants, the used container, the addition of other ingredients such as acids, sugar or milk, and others (57–60). Acidification of the solvent can enhance the extraction; for example, hydrochloric acid, acetic acid, or other acids might be used (34, 60, 61). Moreover, Lajoie et al. (62) summarized and compared the most relevant approaches to enhance the water solvent potential for extracting of natural products.

Different techniques can be used to extract bioactive compounds from the plants and are classified as conventional and nonconventional approaches. These conventional extraction methods are simple, easily available, and low-cost, but their use is limited by the diffusion of solvents into the plant’s cell walls (63). These methods are decoction, leaching, maceration, hydro and steam distillation, percolation, digestion, soxhlet extraction, heat, reflux extraction, and others (63, 64). However, their disadvantages include lower extraction selectivity, a longer time of extraction, and thermal destruction of heat liable chemical compounds at higher temperatures (34, 63). Moreover, these conventional methods might require the use of pure and expensive solvents, which might be toxic. Increasing the temperature enhances the solubility and diffusion coefficient of the phenolic compounds, which speeds up the extraction process (65). However, some compounds may degrade at higher temperatures; for example, rutin begins to degrade at 75°C, and its concentration decreases with increasing temperature until it is no longer quantifiable (90°C) (58, 66). Moreover, the concentration of volatile compounds in the final extract could increase as a function of the temperature, implying that if the water is not hot enough, the aroma from herbs would not be released optimally (67).

On the other hand, non-conventional approaches include ultrasound, subcritical and supercritical fluid, microwave, enzyme, pulsed electric fields, instant controlled pressure drop, extrusion, and other assisted extractions (34, 68). These innovative and promising extraction techniques are used to recover plants’ bioactive molecules while reducing extraction time, energy, and solvent consumption to reduce waste production (68). The advantages of supercritical fluid extraction are that it is suitable for heat-sensitive compounds and prevents their oxidation, ecology without toxic solvents, shorter extraction time, and low viscosity and higher diffusion coefficient (69). That method might extract compounds like flavonoids, carotenoids, essential oils, and fatty acids from natural plant materials; moreover, it might employ several permitted extraction solvents; for example, non-polar hydrocarbons such as propane and butane, carbon dioxide, butane, nitrous oxide, ethanol, etc. (54, 69, 70). On the other hand, supercritical fluid extraction is unsuitable for extracting polar compounds (69). Extraction by the pulsed electric field is ideal for a high number of samples for a short extraction time; moreover, this technique facilitates purified extract, minimizes the degradation of the thermolabile compounds, and increases the extraction yield (69, 71). However, process parameters must be carefully monitored since they depend on various factors such as specific energy input, pulse number, field strength, and temperature (69, 71). Ultrasonic-assisted extraction is a process that extracts target molecules from a mixture of solvents by using ultrasound energy. Furthermore, the benefits of this technology include a higher yield with a shorter processing time and a lower quantity of chemicals and energy (69, 71).

Polyphenols’ structural variety and wide distribution affects their extraction from the complex plant matrix; for example, insoluble polyphenols and flavonoids are located in the cell wall (72, 73). In contrast, soluble phenolic compounds like anthocyanins are found inside the cell vacuoles (72, 74). The high temperature of the solvent may promote the distribution of cell wall polysaccharides into the solvent and weaken the integrity of the cell wall, allowing phenolic compounds to pass into the infusion easily (75, 76). Their extractability could also be influenced by the chemical properties of phenolic substances, such as their molecular structure, polarity, concentration, number of aromatic rings, and hydroxyl groups. Complete extraction of some polyphenols may not be possible due to the formation of complexes with other metabolites, such as proteins and carbohydrates (77–79). Additionally, the extract’s concentration, purification, and fractionation are important methods for phenolic compounds analysis and the subsequent application of the plant extracts in food and beverages (73).

Moreover, it is important to consider that the overall activity of plant extracts may be caused by combinations of constituents with synergistic, additive, or antagonistic effects. For example, high thiamine concentrations (0.1 and 0.8 mg/100 g) reduced the antioxidant activity (AA) of ethanolic tea extracts (80), or Murakami et al. (81) observed a synergistic effect between some phenolic compounds and α-tocopherol.

The current review focuses on using plant extracts to fortify food or beverages, extend their shelf life, control microbiological activity, or increase these products’ sensory and nutritional properties. This study evaluates the potential of aqueous or ethanolic plant extracts rich in bioactive compounds as a natural food additive. In addition, the present paper aims to collect and provide some of the most recent literature on water-ethanol plant extracts and their food applications to present well-arranged data using tables. The application of active plant extracts in oil, beverages, pastry, meat, and dairy products was also studied in detail.

## 2. Application of plant extract in food products

The current trend is a healthy lifestyle and diet; therefore, consumers worldwide demand the development of food with a reduced level of synthetic ingredients like colorings, flavors, stabilizers, preservatives, and other food additives (82, 83). These requirements respond to concerns about the potentially harmful effects of long-term consumption of artificial additives on human health (84). However, only a small percentage of food producers develop products without food additives due to goods’ short shelf life and their susceptibility to microbial contamination. Consequently, there is an effort to find alternative natural food additives with appropriate organoleptic, antioxidant, or antibacterial properties that will extend the shelf life of food products and be attractive to consumers (33, 82, 83, 85).

Lipids are the food ingredients most susceptible to oxidation, which limits the shelf life of products or changes their quality and nutritional value. The most prevalent cause of rancidity is lipid autooxidation, a free radical chain reaction. Heat, light, oxidants, ionizing radiation, metalloproteins, transition metals, enzymes, and compounds susceptible to homolytic cleavage might initiate lipid oxidation. Oxidative changes can result in rancidity, change in flavor and odor, color loss, nutritional value, or toxic compounds that can be harmful to consumer health (hydroperoxides, endoperoxides, and epoxides of fatty acids and cholesterol, alkoxy and hydroxy radicals, aldehydes such as malondialdehyde) (86–88). Consumers notice odors formed from molecules such as hexanal, heptanal, and other volatile compounds produced by lipids’ oxidation, and these substances reduce the shelf life of a food product (89).

Antioxidants can delay the oxidation of lipids in food, which helps in food preservation by slowing the development of rancidity and color change. Natural antioxidants are a heterogeneous group of compounds with various chemical properties. This category includes phenolic compounds such as flavonoids, and the antioxidant activity (AA) is affected by their chemical structure and interaction with biomembranes. Many compounds have AA and might inhibit oxidation, but only some of them can be used in food. According to some studies, plant extracts can contribute to consumers’ health while they might have a more potent antioxidant capacity than synthetic antioxidants (90, 91). On the other hand, several natural antioxidants showed lower antioxidant activities than their synthetic counterparts, suggesting that they should be applied to food in larger amounts (92).

Although the traditional usage of plants supports their safety, and they are generally recognized as safe (GRAS) food additives, many of them have not been studied in scientific research, and their efficacy and safety have not been thoroughly established (93). Assessing their safety and toxicity is important because plants’ extracts are usually concentrated and contain a higher amount of bioactive and toxic compounds. The biological effect of plant extract might be therapeutic, functional, or poisonous and depend on the quantity and quality of the components (94). For example, their application may cause phytotoxicity and allergies in excessive concentrations (85, 94); moreover, some extracted polyphenols in higher doses might contribute to the development and progression of cancer (42). Safety and toxicity studies are important for validating plants’ ongoing use and help establish the dosage of extracts for further application (95, 96).

If plant extract (e.g., herbal infusion) already has a significant history of consumption in food in the EU before 15 May 1997, it implies that this extract does not fall within the scope of the Novel Food Regulation (EC) No. 258/97 and can be considered safe. Also, the type of solvent should be considered because an aqueous extract may be classified entirely differently from a plant extract prepared with other solvents (97). According to López-Rodríguez et al., a total of 18 plant extracts have been approved as novel food or ingredients until 2022 (98). The examples of leaves extract in this group are rosemary extracts (*Rosmarinus officinalis* L., ethanolic extracts or extracts prepared by supercritical CO_2_ extraction) (99), aqueous extracts of *Ilex guayusa* (98), and a purified extract of green tea [*Camellia sinensis* (L.)] Kuntze, containing at least of 90% (−)-epigallocatechin-3-gallate (100). Roots are another part of plants that could be used for the preparation of extracts; for example, the powder of three herbal roots (*Angelica gigas* Nakai, *Phlomis umbrosa* Turcz., and *Cynanchum wilfordii* Hemsley) obtained by aqueous extraction, evaporation, and finally, spray drying (101). Moreover, this powder might be used as a food supplement, and its maximum level is 175 mg daily for adults (101). Other roots used to prepare aqueous-ethanolic extract with maltodextrin are *Panax notoginseng* and *Astragalus membranaceus* (Fisch.) Bunge; in addition, the maximum level of consumption for adults except pregnant women is 35 mg/day (102). Furthermore, cranberry extract powder is obtained by ethanolic extraction of concentrated juice from *Vaccinium macrocarpon* fruit, and the maximum level is 350 mg/day for adults (103).

Quillaia extract (E 999, bark and wood) is probably safe when used in amounts found in food; moreover, an acceptable daily intake of 0–1 mg/kg body weight per day has been determined. Additionally, the presence of saponins in quillaia extract was responsible for the adverse effects on human health (104). In addition, betanin extracted from beetroot was accepted as food additive E162; this natural colorant provides a red-violet color when applied to food (105). The majority of approved application of beetroot extract has no maximum numerical level for a food additive, and only the necessary amount of extract is utilized (quantum satis) (106). However, the application of colors is forbidden in any food products for infants and young children (106).

Green tea is rich in catechins, of which (−)-epigallocatechin-3-gallate (EGCG) is the most relevant and abundant polyphenol (107, 108). However, according to the European Food Safety Authority, green tea extract consumed as a food supplement at higher concentrations of EGCG exceeding 800 mg/day increases the likelihood of liver damage (108). As a result, food with green tea extract must include less than 800 mg of EGCG, and producers must specify on the label that the total daily dose of 800 mg must not be exceeded (100). However, a safe dose could not be established (107). Because EGCG is a polar molecule soluble in water and ethanol-water mixtures (108), adverse effects related to the consumption of green tea were observed on both aqueous and hydroalcoholic extracts (94).

**Figure 2:**
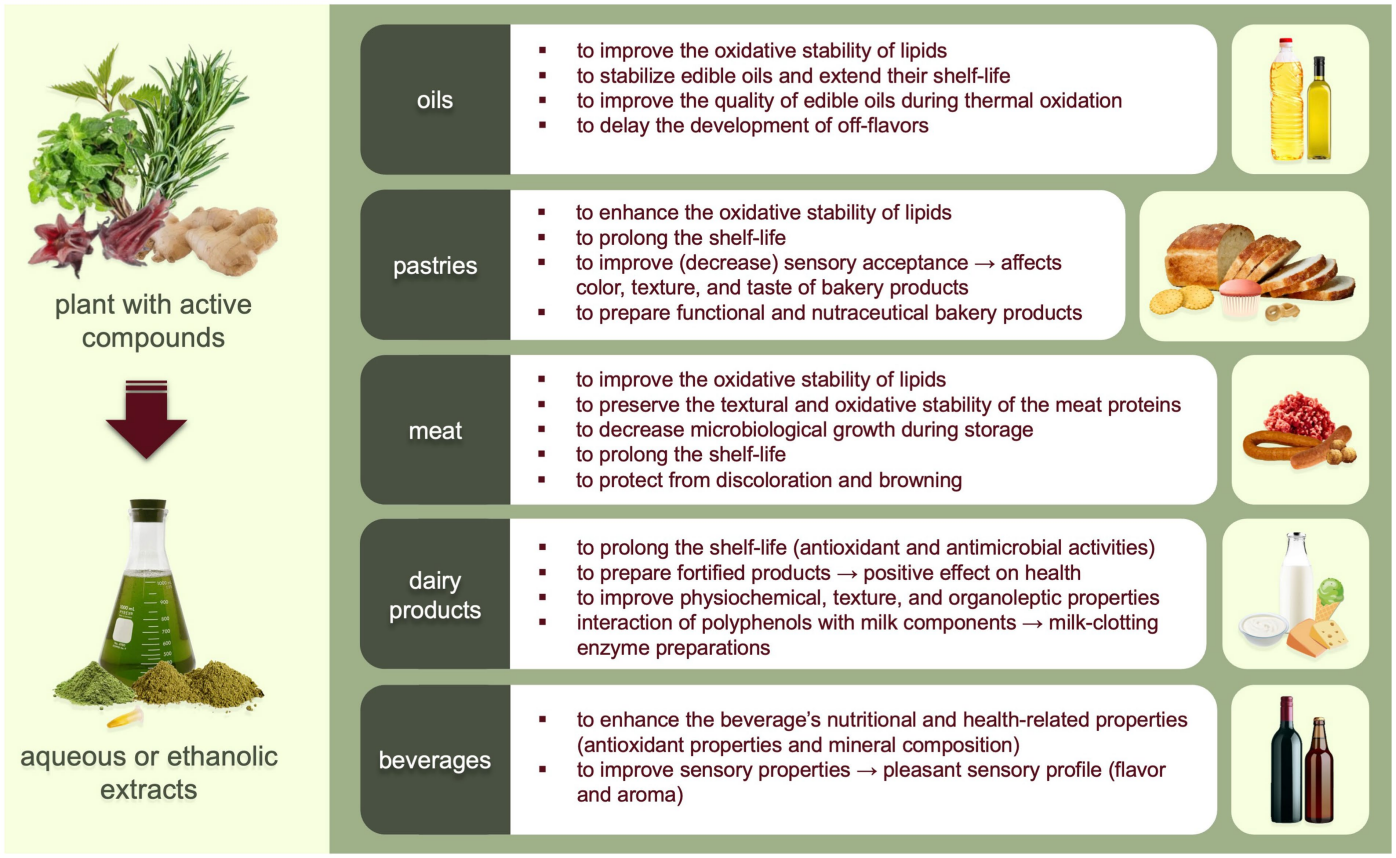
Application of water or ethanol-water plant extracts in food and beverages.

Functional food fortified with plant extracts appear across food categories, as shown in Figure 2. Herbal extracts have the potential to be used in different food products containing fats and oils, including biscuits, fresh meat, meat preparations, cheeses, yogurts, margarine, mayonnaise or salad dressings, and other products (81, 83, 86, 109).

Before food application of extract, it is necessary to consider the type of food matrix to which it will be added because the nature of some natural antioxidants might limit their solubility depending on whether they are hydrophilic or hydrophobic (110). For example, the lipophilic nature of carotenoids might limit their solubility in aqueous-based products (111). In contrast, hydrophilic molecules like phenolic acids cannot be easily incorporated into high-fatty food products such as oils and fats (111, 112). Moreover, adding plant extracts into the food matrix can be challenging since the bioactive compounds are exposed to different processing conditions, affecting their stability (113). For example, phenolic compounds’ molecular size and structural conformation influence their sensitivity during food processing (113). In addition, various degradation mechanisms of bioactive molecules, like oxidation, hydrolysis, condensation, enzymatic deterioration, polymerization, and others, can occur due to the heterogeneous composition of the extract (111, 113).

Incorporating plant extracts as a food ingredient could negatively impact the product’s quality due to their distinctive aroma and color (114). Because plant extracts are usually potent antioxidants, rich in phenolic compounds, they are likely to contribute to the bitter taste, astringency, and darkening of manufactured food and beverages (23, 104, 115, 116). Because of changes in the organoleptic properties of food products, consumers may reject food or beverages containing plant antioxidants, especially those with essential oils (92). Furthermore, these volatile essential oils give a strong flavor, and polyphenols may have an unpleasant taste; therefore, encapsulation methods might mask these compounds before they are added to food (92, 118). For example, liposomes, nanoemulsions, nanoparticles, phytosomes, and ethosomes might be used to protect and improve the stability of plant bioactive molecules (111). On the other hand, another potential strategy for overcoming natural extracts limits is investigating the synergistic and additive interactions between antioxidants, which might result in more effective multi-antioxidant systems with less of each component (110, 118).

### 2.1. Oils

Edible vegetable oils are mainly triacylglycerols, composed of various fatty acids, which are highly susceptible to oxidative processes (119). Plant extracts can be considered potential sources of natural antioxidants for stabilizing edible oils since they prevent undesirable changes during storage and frying operations and might extend the oil’s shelf-life. As mentioned above, the hydrophilic character of phenolic acids may limit their potential application as antioxidants in oils; however, modifying their chemical structure by incorporating them into triacylglycerols might enhance their solubility and broaden their use in emulsions (112). Lipid oxidation is one of the most significant issues during the frying and storage processes, which results in the formation of free radicals, the loss of food quality, and it could cause illness (120, 121).

According to Soleimani et al. (122), the aqueous leaf extract of *Hyssopus officinalis* L. and aerial parts extract of *Echinacea purpurea* L. reduced the oxidation rate of soybean oil exposed to 70°C for 5 days. All samples containing 200 to 1,000 ppm of hyssop extracts were more stable than the control soybean oil when evaluating peroxide and thiobarbituric acid changes, as presented in Table 2. The AA of these samples was comparable to the synthetic antioxidants butylhydroxytoluene (BHT) and butylhydroxyanisole (BHA) at 100 ppm. Both extracts could be appropriate alternatives to synthetic antioxidants, having no additional limits to get optimal effects. Pourashouri et al. (125) found that the concentrated aqueous extracts of wild pistacia, green tea, and rosemary extended the stability of a 10% fish oil-in-water emulsion for a few days.

**Table 2:** Application of plant extracts in oil.

Plant	Extract	Application	Concentration	Reference
*Hyssopus officinalis* L. *Echinacea purpurea* L.	Water extract – leaves of roselle and aerial parts of echinacea (preparation was not specified)	Natural antioxidants in soybean oil (reduction the oxidation rate)	200, 400, 600, and 1,000 ppm, positive control BHA, BHT at 100 and 200 ppm	(122)
*Rosmarinus officinalis* L.	5 g of lyophilized rosemary to 100 mL of ethanol, extraction (16 min at 25°C), filtration, solvent removal (40°C), stock solution preparation (1:10 - ethanolic extract per ethanol)	Control of thermooxidation of soybean oil during heating	3,000 mg of rosemary extract/kg, positive control of 50 mg of TBHQ/kg, and extract mixture with TBHQ were evaluated	(123)
*Rosmarinus officinalis* L.	Dried rosemary powder was extracted using a soxhlet apparatus (2 h) with ethanol as solvent, 1/10 ratio (powder/solvent), filtration, evaporation	Improvement of sunflower and soybean oils quality in frying conditions	800 mg extract to 1 kg of a mixture of oils - sunflower and soybean (50:50)	(124)
*Rosmarinus officinalis* L. *Pistacia atlantica*	20 g of dried leaves with 200 mL of water boiled at 100°C for 15 min, filtration, evaporation	Plant extracts’ influence on the oxidative stability of fish oil emulsion	Were evaluated in 10% fish oil-in-water emulsions	(125)
*Urtica dioica* L. (seed) *Salvia officinalis* L. *Mentha arvensis* L. *Rhus coriaria* L. Thymus vulgaris	Freeze-dried extract: soxhlet extraction of 20 g of dried sample with ethanol: water solution (80:20), evaporation (40°C)	Corn oil oxidation inhibition	1 g of extract per kg of stripped corn oil; positive control BHT and ascorbyl palmitate	(126)
*Rosmarinus officinalis* L.	1:20 (w/v) dry leaves powder to ethanol solution as solvent (80%), ultrasonic bath at 50°C (40 KHz, 0.5 h), filtration, oven evaporation (ca 30 °C) and lyophilization	The application of rosemary extracts to increase the oxidative stability of flaxseed oil rich in omega-3 fatty acids	200 ppm, positive control: 100 ppm α-tocopherol, and 200 ppm BHT	(127)

Aqueous extracts from aromatic herbs such as sage, sweet marjoram, and dittany showed a protective ability against lipid oxidation in refined sunflower oil. This ability to slow the oxidation of lipids is probably due to the high level of polyphenols, which might actively eliminate active oxygen and suppress its negative effects (128).

Casarotti and Jorge (123) studied the thermal stability of soybean oil containing rosemary extract over 20-h heating at 180°C. The addition of rosemary extract increased the oxidative stability of oil and resulted in a lower production of total polar compounds. Moreover, this extract showed a higher antioxidant potential compared to the synthetic antioxidant tertiary butylhydroquinone (TBHQ).

According to Wang et al. (127), the addition of rosemary extracts into omega-3 fatty acid-rich flaxseed oil might positively delay lipid oxidation and the oil’s oxidative stability (92). Phenolic constituents such as carnosol, carnosic acid, and rosmarinic acid were detected in rosemary extract (124, 129). These compounds might affect the oxidative stability of flaxseed oil during storage due to the synergistic interaction of naturally occurring tocopherol with potential pro-oxidant activity (127). In addition, Chammem et al. (124) successfully applied rosemary extract into the oil mixture to inhibit oxidation throughout frying. This extract can slow down oil oxidation throughout its use in frying. Oil treated with extract conserves more unsaturated fatty acids after 30 h of heating, suggesting its resistance against oxidation. A pleasant flavor was detected in oil treated with rosemary extract during frying.

Rosemary extract (E392) might be used in various water-free fats and oils. In non-heat-treated vegetable oils with a polyunsaturated fatty acid concentration higher than 15% (w/w) of the total fatty acid, the maximum allowed limit is 30 mg/L of the sum of carnosic acid and carnosol (130). This food additive is used commercially in pumpkin (MVDr. Jiří Pantůček - TopVet, China), linseed (Green idea s.r.o., Belgium), avocado (Allnature s.r.o., Czech Republic), milk-thistle (MVDr. Jiří Pantůček - TopVet, Czech Republic), and rice oils (Allnature s.r.o., Czech Republic). Other applications of the food additive E392 are fish and algal oils; frying oils and fats; beef, poultry, sheep, and porcine fats; lard; oils and fats used in the professional production of heat-treated meals (130). Other applications of the food additive E392 are fish and algal oils; frying oils and fats; beef, poultry, sheep, and porcine fats; lard; oils and fats used in the professional production of heat-treated meals. Furthermore, the maximum permitted limit (MPL) for carnosol and carnosic acid in these products is 50 mg/L (130). Examples of commercial items are fish oil Neuro treska (Nom oils, Czech Republic), algae oil capsules (Kala Health, Netherlands), and professional sunflower oil for frying (Olitalia, Italy). Moreover, rosemary extracts cannot be applicated to virgin, olive, and pomace oils (130).

### 2.2. Pastries

Maintaining the quality of baked goods such as biscuits is important from an economic point of view because they are stored for a longer period before consumption. We can find a few studies dealing with adding plant ethanol-water extracts rich in phenolic compounds to biscuits to increase their shelf life, detailed in Table 3 (132, 133, 140). The natural extracts made from raisins (*Vitis vinifera*) and drumstick leaves (*Moringa oleifera*) had a stronger activity than the synthetic antioxidants BHA. Biscuits prepared with 1–2% of these extracts could be stored over six weeks because peroxide and free acids values were lower than in control and BHA-treated samples (131). Caleja et al. (133) found that the addition of synthetic BHA additives or natural extracts gave similar AA to biscuits.

**Table 3:** Application of plant extracts in pastries.

Plant	Extract	Application	Concentration	Reference
*Moringa oleifera Vitis vinifera*	100 g of plant material with 50% (raisins) or 90% ethanol solutions (drumstick) - shaking (6 h) and filtering; then 1-h shaking of dried residues after filtration with 90% ethanol (two times), filtration; the mixture of extracts was evaporated	Improvement of oxidative stability and sensory acceptance of biscuits by plant extract	1% drumstick, 2% raisin, 200 ppm synthetic antioxidant BHA	(131)
*Hibiscus sabdarifa* L.	100 g of overnight soaked fine powder (0.55 mm) and 450 mL water (overall 650 mL water) was heated at 80°C for 1 h, filtration	Functional cupcakes with health benefits and sensory acceptable (color benefits)	50 g to 250 g of dough (20%)	(132)
*Matricaria recutita* L. *Foeniculum vulgare* Mill.	Lyophilized decoction – boiled 5 g of dried powdered plant and 200 mL of water (for 5 min), filtration	Improving the AA of biscuits without significant color or nutritional changes	80 and 800 mg, a positive control of 80 mg of BHA	(133)
*Clitoria ternatea* L.	Commercial spray-dried extract (flower petal)	Sponge cakes fortified with colorful extracts; physicochemical characteristics (color, total phenolics, AA, water activity), texture attributes, and sensory acceptability	5, 10, 15 and 20% (w/w)	(134)
*Vitis vinifera* ‘Barbera’	Spray-dried extract—125 g grape pomace powder with 1 l of 60% aqueous ethanol solution was continually stirred at 60°C for 2 h before centrifugation, evaporation, and spray-drying with maltodextrin	Evaluate the effect of encapsulated extract on the functional characteristics and sensory acceptance of biscuits	1.2, 2.3, and 3.5% on dough weight	(135)
*Melissa officinalis* L. *Hyssopus officinalis* L. *Urtica dioica* L.	10 g of dried leaves and 250 mL of aqueous ethanol (70% v/v) were heated at 45°C for 10 h before filtration, and evaporation (40°C)	Shortbread cookies enriched with herbal extracts and their influence on chemical and sensory features (color intensity); antioxidant activity and fat quality after three months of storage at room temperature	0.02, 0.1, and 0.2%, positive control: 0.02% BHA	(136, 137)
*Olea europaea* L. (leaves)	An ultrasonic bath (39 kHz, 200 W, 1 h, 25°C) was used to extract 0.5 g of dried powder with 10 mL of 50% (v/v) ethanol solution, followed by filtering	Enriched taralli with phenolic-rich olive leaves extract; nutritional content and bio-accessibility of the main phenolic components (*in vitro* digestion)	75 g to the dough	(138)
*Mentha spicata* L.	2.5, 5.0, or 7.5 g of dried leaves were soaked for 10 min in 100 mL of hot water (90–99°C), then filtered	Fortified bread with increased antioxidant content; sensory evaluation	100 mL	(139)
*Citrus limon* (L.) Osbeck	20 g ground freeze-dried lemon pomace and 200 mL 50% ethanol solution (v:v) were mixed in an ultrasonic homogenizer at 40°C for 40 min, followed by centrifugation, filtration, and dilution to 200 mL with the extraction solvent	Biscuits with improved lipid oxidation resistance; higher quality in terms of functional, nutraceutical, and sensory acceptability (flavor, aroma, and appearance)	50 mL of extract	(140)

In addition, Kozlowska et al. (136) prepared cookies by adding extracts of hyssop, nettle, or lemon balm and substituting some of the palm fat with walnut oil (cold-pressed). Although including hyssop or lemon balm extracts in the formulation of cookies may increase their quality and appeal to consumers, synthetic antioxidant BHA (0.02%) was still a more efficient inhibitor of lipid oxidation than herbal extracts (136).

A few authors have dealt with the use of natural extracts obtained from by-products such as lemon (140) or grape pomace (135, 141) in biscuits. Biscuits enriched with ethanol extract from citrus lemon pomace showed higher antioxidant activity and phenolic content and improved nutraceutical properties than the control sample (140).

Plant extracts can be used as additives in bakery products, including muffins, cakes, bread, and snacks (taralli – round breadsticks). The addition of roselle extract to cupcakes enhanced their ash, fiber, ascorbic acid, and total anthocyanins content, and the last one caused the color of the muffins turned pink (132). The addition of *Clitoria ternatea* extract also changed the final product’s color, increased total phenolic content (TPC) and AA, and at the same time, inhibited lipid peroxidation in the sponge cake. The hardness, adhesiveness, gumminess, and chewiness of the cake increased as the amount of extract in the cake was higher, whereas the cohesiveness, springiness, and resilience decreased (134). Furthermore, Nedamani (142) prepared cakes containing *Berberis Vulgaris* extracts, lycopene and chlorophyll oleoresins (tomato, spinach) as natural colorants. The panelists considered that colored cakes are more interesting for consumption than non-colored cakes. However, cakes made with *Berberis vulgaris* extract got the lowest color, taste, and odor scores, while texture and porosity were comparable to other samples. The novel product taralli with olive leaves extract had better nutritional quality, increased TPC, TFC, and AA. The sensory aspect was comparable to the control sample (138).

Several studies have looked at adding plant extracts to bread made from flaxseed hull, barley hull (143), green parts of the Tartary buckwheat (144), grape seeds (145) or spearmint (146). Fortified bread with spearmint extract had higher TPC, DDPH radical scavenging activity, and ferrous-ion chelating (FIC) ability than the control bread. The strong taste of spearmint may account for the decrease in sensory scores (146).

Extracts may be applied to various types of items containing cereals like fine bakery wares (MPL 200 mg/kg or 2,000 mg/kg), fillings of stuffed dry pasta (MPL 250 mg/kg), noodles (MPL 2,000 mg/kg), batters (MPL 12,000 mg/kg), soda bread and rolls (MPL 20,000 mg/kg) (130). Moreover, rosemary extract may be used in products like whole grain crispbread (Celpo spol. s r.o., Slovakia), spaghetti carbonara (HFC GmbH, Germany), pizza (Dr. Oetker, Germany), buns with chocolate-flavored filling (Tims Foods Ltd., Bulgaria), and cookies with rye flour (Rej s.r.o., Czech Republic). Another application of antioxidant E392 is whole grain biscuits (Pečivárne Lipt. Hrádok s.r.o., Slovakia), which contain extracts from roasted barley, rice, and chicory. In addition, the pretzels (Perfetti Van Melle Czech Republic, s.r.o., Czech Republic) include rosemary extract, barley malt extract, and paprika extract (coloring). Additionaly, beetroot extract (E162) is used in carrot cake cupcakes (Wicked, United Kingdom), burritos (Old El Paso, France), snuggles (Snack Day, Germany), birthday cakes (Fibre One, United Kingdom), and familial biscuits (Mini BN. France).

### 2.3. Meat

Meat and minced meat are highly susceptible to oxidation and microbial contamination due to their high-fat content, unsaturated fatty acids, moisture, texture, and lack of heat treatment (23). During the meat processing and storage process, several changes occur, forming several reactive and toxic compounds. Oxidation of meat products can change the color, aroma, taste, texture, and appearance of the product; hence, sensory qualities are the most critical factors for determining the oxidation level and shelf life of meat (147). The use of plant extracts in meat products has received interest due to their beneficial natural antioxidant and antibacterial properties. Table 4 shows the application of various plant extracts as antioxidants and possible antimicrobial agents that benefit several meat products.

**Table 4:** Application of plant extracts in meat.

Plant	Extract	Application	Concentration	Reference
*Moringa oleiferia*	A concentrated aqueous extract (soxhlet extractor, 18–20 h) made from 100 g of dried herbs with 600 mL of water, drying under reduced pressure at 40–50°C	Plant extract with high AA used as an antioxidant in precooked refrigerated goat meat patties (reduce lipid peroxidation, not affect sensory attributes)	0.1%, positive control 0.1% BHT	(148)
*Aspalathus linearis*	Concentrated water extract (spray drying)	Rooibos extract as a natural food preservative in jokbal (pig’s trotters); antimicrobial activity and inhibition of lipid oxidation	10% (w/w)	(149)
*Humulus lupulus* L.	infusion: 2 g of hop pellets were boiled with 50 mL of water for 2 min; then the extract was filtered	oxidative stability of lamb patties during storage (stability of color, lipids, and proteins); sensory acceptance	2 g hop infusion per 1 kg of patties	(150)
*Liriope platyphylla Saposhnikovia divaricate Akebia quinata*, *Lonicera japonica Chelidonium majus*	Dried powder with distilled water (in a ratio 1:10) was refluxed for 6 h at 80°C, residues and water (1:5) at 80°C for 2 h, filtration, dried under vacuum (< 40°C) and freeze drier	Improve the oxidative stability, and quality of emulsified pork sausage	0.2%	(151)
*Perilla frutescens* var. acuta	50 g of dried leaves and stems with 1.95 l water were extracted in a shaking bath at 70°C for one day, then centrifugation, filtration, and lyophilization	Enhance the shelf life and sensory parameters of cooked beef patties (antioxidant and antimicrobial potential)	0.6%	(152)
*Plinia jaboticaba* (Vell.) Berg, genotype Sabará	Dried powdered peels and 95% ethanol (12:1) were stirred at room temperature for 1 h, followed by filtration, re-extraction of residues by 95% ethanol (twice), filtration, evaporation, volume adjusted to 50 mL with water	Improve bologna-type sausages’ shelf life, oxidative stability, sensory and microbiological qualities	0, 0.25, 0.5, 0.75, and 1%	(153)
*Urtica dioica* L.	Maceration of 2 or 4 g of dried leaves in 100 mL of water for 1 h at 60°C, and filtration	Improve lipid and color stabilities of cooked pork sausage	0, 300, and 600 ppm	(154)
*Rosemarinus officinalis Eugenia caryophyllata*	50 g of dried powder was extracted into 400 mL of 95% (v/v) ethanol under constant shaking for 12 h; residue after filtration was re-extracted with 200 mL of 95% ethanol for 12 h, followed by filtration, evaporation, and freeze-drying of the extract; before application - extracts were dissolved in water	Determined the antioxidant, antimicrobial, and sensory effects of spice extracts on raw chicken conservation stored in refrigeration (shelf-life improvement)	Concentrations: 1% rosemary extracts, 1% clove extract and mix 0.5% from each extract	(155)
*Betula pendula*	Dried grounded leaves were extracted in ethanol solutions containing 50, 75, and 90% ethanol at a 1:30 (w/v) ratio at ca 4°C for one day in the dark under continual stirring, followed by filtering, evaporation, and freeze-drying	Lowered beef patties’ discoloration and browning and considerably delayed lipid degradation	0.1 and 0.3% (w/w) lyophilized extracts to 20 g beef patties	(156)
*Thymus vulgaris* L.	100 g of dried plant material with 250 mL of 80% ethanol at room temperature and overnight (repeated 3× with new ethanol), evaporation (50°C), freeze-dried	Lipid stability and protein digestibility (nutritional value) of frozen ground pork meatballs	0.05%	(157)
*Perilla frutescens*	One hour of reflux extraction of 10 g of dried herbs with 60 mL of 95% ethanol, residues after filtration was re-extracted twice with 30 mL of ethanol solution (95%), then extract was evaporated (40°C) and freeze-dried	Extended shelf-life of surimi fish balls enriched with herbal extract; reduced lipid and protein oxidations and decreased microbiological growth during storage	0.03%	(158)
*Curcuma longa* L.	80 g of dried, grounded turmeric roots and made to 290 mL supercritical fluid extraction CO_2_ (flow rate of 10 g/min) with ethanol like cosolvent (flow rate of 15 g/min), static time: 20 min, dynamic time: 5 h, pressure: 300 bar, glass beads size: 3 mm	Improved sensory attributes and oxidative stability of fresh lamb sausages with turmeric extract	250, 500, and 750 mg/kg	(159)
*Syzigium aromaticum* L.	Dried powdered clove with distilled water in the ratio of 1:5 (w/v) for 6 h at 90°C; leftovers were extracted under the same condition but twice as long, then the extract was filtrated and evaporated (90°C)	Improvement of the oxidative and sensory stabilities (color, odor, lipid, and protein stability) in cooked beef patties at refrigerated storage	0.1% extract, positive control 0.02% BHT	(147)

The type of meat influences its oxidation stability because red and darker meat contains a higher concentration of heme protein myoglobin (160), which is primarily responsible for raw meat’s red color, and redox reactions on the iron atom cause color changes (86). During meat storage and processing, ferric heme (hemin) might dissociate from the heme-globin complex; consequently, released hemin might be a primary promoter of lipid oxidation and play an essential role in the catalysis of lipid peroxidation (161, 162). Additionally, reactive oxygen species and heme oxygenase might destroy the heme ring and release iron from it (161).

Green tea extract can increase the color fastness of fresh meat products (163). The vermilion color of meat is caused by oxymyoglobin, whereas the brown color is caused by metmyoglobin, which is formed by the oxidation of oxymyoglobin (86). Green tea extract contains catechins, which show high AA in chicken and beef (163).

The addition of antioxidants delays lipid oxidation, inhibit oxidative transformation, and improve meat quality. Natural antioxidants can contribute significantly to developing functional meat products with higher nutritional and health advantages, extended shelf life, and superior product quality. For example, ethanol extracts from rosemary and thyme (157) and clove concentrate (147) were added to minced meat as natural preservatives. Lyophilized extracts from fenugreek seeds and ginger rhizomes added to the beef patties showed vigorous AA comparable to commercial antioxidants. These lyophilized extracts can inhibit lipid oxidation during cold storage, preventing discoloration and forming a rancid odor and thiobarbituric acid (164).

In other studies, they applied plant extracts in meat patties, for example, *Betula pendula* (145), *Moringa oleiferia* (156) and *Humulus lupulus* L. (148). According to Azman et al. (2017), a reduction of lipid oxidation was observed in the meat samples with *Betula pendula* extract, followed by a lower concentration of metmyoglobin and prolonged color stability of meat. The extract demonstrated the ability to delay the lipids degradation present in the muscle of the meat (156). The incorporation of clove extract in preparing cooked beef patties resulted in a significant decrease in thiobarbituric acid reactive substances (TBARS) value, carbonyl content, and enhancement of the stability of sensory attributes. It was used to hinder protein and lipid oxidation (147). The moringa extract had higher TPC and was more effective than BHT in keeping pre-cooked chilled goat meat patties at a low TBARS level which suggests that lipid peroxidation was slowed (148). Furthermore, hop infusion increased raw patties’ lipid and color stability during refrigerator storage, albeit to a lower extent than sodium ascorbate (reducing agent). Consumers preferred patties with hop infusion over those with hop powder (150). Beef patties treated with perilla extract had higher sensory scores than control samples; also, this meat product may have increased shelf life by inhibiting the growth of *Escherichia coli* and aerobic bacteria during the storage period (152).

In addition to beef, chicken, mutton, and goat meat, herbal extract was also used in fish meat. Surimi fish balls were fortified with perilla extract, which improved the product’s shelf-life. It slowed lipid and protein oxidation during storage at 4°C, reducing TBARS values and protein carbonyl contents (158). Furthermore, plant extracts of grape seeds, pomegranate rind (165), and rosemary (166) were added to the minced fish. These extracts have the potential to be used as a natural antioxidant in raw minced fish tissue to slow oxidative changes and extend frozen storage time (165, 166).

Another potential use of plant extracts is in salami, sausages, and other charcuterie products. According to de Carvalho et al. (159), turmeric extract which can substitute the synthetic antioxidant sodium erythorbate (500 ppm), increased the AA of fresh lamb sausages. The addition of 0.5%–1.0% jabuticaba extract, which had a strong antioxidant effect, did not influence the sensory quality of bologna-type sausages and might prevent the decrease of their sensory acceptability during storage for 35 days (153). Furthermore, Baldin et al. (2018) combined mortadella sausage with microencapsulated Jabuticaba water extract (*Myrciaria cauliflora*) rich in colorful anthocyanins (167). This addition of extract improved taste and texture acceptability; however, color, aroma, and pH value were reduced (167). Latoch and Stasiak reported that adding aqueous nettle extract (600 ppm) decreased TBARS value and increased the color stability and sensory parameters (overall quality, flavor, odor) in sausage (154).

Jones et al. (168) used rooibos (*Aspalathus linearis*) extract to increase the oxidative stability of game droëwors, a dried meat sausage produced from blesbok and springbok meats combined with beef meat to improve fat content. The concentration of rooibos extract 1.0% was effective in slowing down lipid oxidation, and with increasing extract concentration, the lipid oxidation level significantly decreased. Moreover, a mixture of 10% rooibos, 4% potassium lactate, and sodium diacetate considerably reduced the amount of *Clostridium perfringens* in pig’s trotters at 10°C. *Aspalathus linearis* has the potential to be a natural preservative because it prevents lipid oxidation, which inhibits the growth of bacteria *C. perfringens* as well as the outgrowth and germination of their spores (149). Adding herbal extracts might be an alternative to the reduced salt content in meat products; for example, the application of basil extract increased the salty taste of low-sodium hams (116). Another application of plant extract could be as a natural colorant (105, 167); for example, beetroot extract and powder (freeze-dried) might be used as alternatives to carmine in sausage processing. Moreover, this extract addition positively affected the sausage’s overall acceptance, appearance, color, and flavor (105).

Only a few studies investigated synergistic effects between plant extracts and essential oils on meat products (169, 170). For example, the combinational effects of aqueous grape seed extract (0.08%, 0.16%) and cinnamon essential oil (0.02%, 0.04%) slowed down lipid oxidation, extended the shelf life, and improved sensory qualities such as color and odor of the cooked chicken sausage (170, 171). Additionally, thymol and carvacrol were the bioactive compounds responsible for the synergistic effect (169). In another study, they prepared pork meatballs and applicated aqueous cranberry and pomegranate extracts (2% w/v), oregano and thyme essential oils (0.150 μg/g), either alone or in combination. The combinations of these natural preservatives could replace synthetic antioxidants in a minced pork meatball with equivalent antimicrobial and antioxidant activities (169). Furthermore, the synergistic interaction was observed between organic acids such as malic and citric acid (plant extracts) and thymol, p-cymene, and carvacrol (essential oils) (169).

The concentration of extract with antioxidant activity in meat products was restricted in a few studies due to the deterioration of organoleptic qualities, the emergence of off-flavors, and the bitterness of the product (23, 172, 173). This may occur due to the interaction of phenolic compounds with other meat components such as lipids, mineral elements, and proteins in muscles (23, 172, 173). Furthermore, plant extract’s antibacterial effect depends on the amount of bioactive molecules; excessive concentrations of these compounds affect negative organoleptic features, which limit their application in food due to the impact on food acceptability (110, 172).

Plant extracts are increasingly being used to substitute nitrites, mostly by utilizing plants such as radish, beetroot, tarragon, celery, lettuce, mint, spinach, basil, and other plants rich in natural nitrites (174). Adding these natural nitrite replacers could help produce meat products with the typical pink color of preserved meat and comparable levels of microbiological safety (175).

In the food industry, spice extracts obtained by distillation in the form of essential oils or oleoresins can be applicated. However, their use necessitates precision in dosing and sufficient dispersion (176). In addition, plant extract could be dispersed on a solid carrier, which preserves natural plants’ flavor and taste while allowing for easy usage and high microbiological purity (176). Furthermore, rosemary extract can be used in non-heat and also in heat-treated processed meat like dried sausages (MPL 100 mg/kg), dehydrated meat (MPL 150 mg/kg), breakfast sausages (MPL 5,000 mg/kg), frozen and deep-frozen mollusks and crustaceans (MPL 5,000 mg/kg), and processed fishery products (130). Rosemary extract (E392) may be used in a variety of meat products, including fried chicken nuggets (Carnj Societa Cooperativa Agricola, Italy), pork spread (Uzeniny Pbram, a.s., Czech Republic), pork sausage csabai (Kaiser Food Kft, Hungary), sausage Pickstick (PICK Szeged, Hungary), sausage Chorizo Artesano (Indústrias Carnicas Navarra, S.A., Spain), sausage Paris (Pick Szeged, Hungary), and turkey sausage (Embutidos Carchelejo, Spain). Extract of beetroot is used as food colorant E162; one of the most popular applications is into sausage; for example, salami Bolzano (Aubel, Belgium), Salami FinBec and garlic salami (Boni, Belgium), Salami (Delifin, France), and original snacks saucisses (Isla Mondial, France).

### 2.4. Dairy product

Enriching dairy products with herbs or spices can help provide functional products with nutritional and healing properties. The main problem with using herbs in cheese production is the possibility of microbial contamination on their surface. Therefore, scientific research is currently focused on developing new methods for the use of natural extracts as preservatives (177). Plant extracts with antioxidant or antibacterial activities have been used in dairy products, for example, to enhance the appearance and overall attractiveness (add color, flavor, and taste) for consumers and improve the shelf life, concentration of antioxidants, and total phenolics of food. Table 5 provides an overview of the current applications of aqueous or ethanolic plant extracts in dairy products.

**Table 5:** Application of plant extracts in dairy products.

Plant	Extract	Application	Concentration	Reference
*Hibiscus sabdariffa* Linn	Aqueous extract: 20 g of dried flower powder and 100 mL water were heated at 60°C for 30 min	Improvement microbiological, antimicrobial, chemical, and sensory characteristics of koumiss probiotic goat milk with roselle extracts	0, 0.5, and 1% (v/v)	(178)
*Cedrus deodara* (Roxb.) Loud	Dried powder was mixed with water in concentrations of 2.5 and 5.0%; these mixtures were stirred for 2 h and refrigerated overnight before filtration	Natural extract as a novel preservative and antimicrobial agent in Kalari cheese; with improved oxidative stability, storage, and sensory qualities	Cheese spraying or dipping (the used amount of extract was not specified)	(179)
*Inula britannica* L.	20 g of dried herbs and 200 mL of water were incubated at 60°C for 1 day, then filtered and freeze-dried	Fortified Cheddar-type cheese with herbal extract; evaluated antioxidant, physicochemical and sensory characteristics (texture, taste, odor, color)	0, 0.25, 0.5, 0.75, and 1% (w/v)	(180)
*Hibiscus sabdarifa* L.	Cold water extraction: 100 g of roselle leaves were soaked in 1 l water for 12 h (in refrigerator), then drained	Preparing functional yogurt with incorporated bioactive compounds to improve the product’s shelf life, and sensory properties	0, 0.2 and 0.4%	(181)
*Rosa* sp. damascena	100 g of fresh petals with 500 mL of water were extracted at 35°C for 1 day, then filtered, evaporated, and dried in an oven at 45°C	Yogurt with a positive effect on health with prolonging shelf-life; qualitative, antimicrobial, and sensory characteristics	2, 4 and 6%	(182)
*Hibiscus sabdarifa* L.	Lyophilized extract: 20 g of dried flower powder and 100 mL of water were pasteurized at 63–65°C for 30 min before filtering, evaporation, and freeze-drying	Evaluate the anti-diabetic potency, as well as chemical, physical, and microbiological properties of probiotic goat-milk yogurt with roselle extract	1%	(183)
*Citrus sinensis*	Dried macerate: 5, 10, 15, 20, 25 g of dry orange peel powder was soaked in 100 mL water for one day while stirring, then filtered and freeze-dried	Functional yogurt with encapsulated orange peel extract; physiochemical, texture, and organoleptic properties	Various ratios of encapsulated extracts were added to skim milk products to obtain 300, 600, and 900 mg polyphenolic compounds	(184)
*Curcuma longa Salvia officinalis Origanum majorana*	Aqueous extract: pulverized plant material (rhizome, leaves) and 1 l of hot water were mixed for 15 min 10% (w/w), they were infused for another hour at room temperature, and filtered	Fortification of skimmed-milk yogurt and UF-Kariesh cheese with herbal extracts; antioxidant activity, microbiological quality, physicochemical and organoleptic attributes	1, 2, or 3% (w/w)	(185)
*Carthamus tinctorius* L.	Ethanol extract: 100 g of dried flowers were shaken for 15 h at room temperature in 900 mL ethanol (99.9%), and filtration aqueous extract: 100 g of dried flowers and 900 mL water (100°C) were extracted for 1 h (reflux condenser), filtration, and freeze-drying	Functional yogurt with improved health benefits, specifically effective in the AA and inhibition effect on enzyme (α-glucosidase, pancreatic lipase)	0.1, 0.5, and 1.0%	(186)
*Solenostemma argel* Hayne	2 g of dried leaves powder with 200 mL of ethanol solution (39.14%, v/v); ultra-sound-assisted extraction (40 kHz, 110 W, 60°C and 37.07 min - two times), filtration, evaporation (50°C), and freeze-drying	Developed a functional yogurt with improved physicochemical properties, increased AA, lactic acid bacteria count, and sensory attributes during cold storage	0.1 and 0.2 g/100 mL	(187)
*Mentha pulegium* L.	10 g of pulverized pennyroyal with 100 mL of ethanol-water solution (50%) were extracted at 45°C for 20 min, filtration, evaporation	Influence of extract or microencapsulated extract on the physicochemical and sensory parameters of probiotic yogurt; viability of probiotic bacteria during storage	750 and 1,000 ppm of extract and nano-extract	(117)
*Portulaca oleracea*	10 g of dried and milled aerial parts of the plant were soaked in 100 mL of water, evaporation	Fortified yogurt with purslane extract and its effect on physicochemical and sensory characteristics for 21 days storage	0, 0.5, 1, 1.5 and 2%	(188)
*Morus alba*	Dried leaves (< 50 mm) were soaked in water (8% w/v) and treated with an enzyme; aqueous extract was produced *via* ultrasound-assisted water extraction for 1 h at 50°C, followed by centrifugation	Enhance the nutritional value and bioactive properties of cottage cheese; was evaluated chemical, antioxidant, healthy qualities, and stability of the bioactive components (*in-vitro* digestion)	1 g to 20 g of cheese	(28)
*Illicium verum Psidium guajava Curcuma longa*	10 g of dried leaves were mixed with 100 mL of water and extracted at 70°C for 12–18 h before centrifugation	Develop cheeses with plant extracts and fish collagen that have anti-ACE properties and proteolytic activity	herbal extract (3 mL) was added to the coagulum curd during cheese preparation	(146)
*Cinnamomum cassia*	Lyophilized extract: dried leaves residues and water 10% (w/v), at 130°C for 1–3 h, centrifugation, freeze-drying	Yogurt fortification herbal extracts and their effects on physiochemical and chemical properties and stability of bioactivity (antioxidant and anti-inflammation activities)	0.8% (w/v)	(189)

Due to their higher moisture content, microbial contamination is more common in soft cheeses than in hard or semi-hard cheeses. Bacterial contamination can occur during technical processing owing to higher handling during manufacture, harmful microorganisms in cheese cultures, or inadequate cooling throughout the production till consumption. The presence of pathogenic microbes and molds may reduce the shelf life of cheese, but they also pose a health concern to consumers. The objective is to minimize these health risks due to the microorganisms with minimal physical, chemical, and organoleptic changes (33, 82).

For example, pine needles extract (*Cedrus deodara* (Roxb.) Loud.) improved lipid oxidative stability by significantly lowering the TBARS and free fatty acid values (179). This cedar extract improved sensory quality while storing low-fat hard and dry cheese “Kalari” and may be commercially exploited as a natural cheese preservative (179). Additionally, fermented cottage cheese with *Morus alba* had superior nutraceutical properties, and the extract had high antioxidant activity DPPH (28). Adding *Inula britannica* extract increased the protein and ash contents of cheddar-type cheeses, with concomitant decreases in the total solids, fat content, and pH. The AA decreased with increasing storage time, correlated with the decreasing content of phenolic substances. The decreases could be related to fermentation and maturation, during which phenolic compounds could interact with milk proteins. Furthermore, *Inula britannica* extract fortification gave a more desirable texture to the cheeses and uplifted their ratings for odor and taste (190, 191). Some cheeses treated with plant extracts showed a higher content of protein, ash, and total polyphenols than the control, as well as decreased pH, fat, and total dry matter values. The addition of the extract probably caused the polyphenols to interact with the milk protein, leading to protein precipitation and cross-linking, and a higher amount of whey was released from the cheese matrix (180, 192, 193). A similar trend of increased protein yield in cheeses treated with extracts was observed after the addition of an aqueous dehydrated green tea extract (194) and ethanol-water extract from grape skin (192). Adding plant extracts rich in phenolics can affect cheese production kinetics, as the change in pH, structure, and source of polyphenols affect the curd’s coagulation and thus the resulting product’s firmness (195, 196).

Fermented dairy products, such as yogurt and milk, are a great matrix for adding plant extracts containing bioactive substances and preparing functional food. Herbal extracts increase these dairy products’ bioactive compounds and AA, further improve color, the sensory and nutritional characteristics, and finally improve microbial stability during refrigerator storage (83, 197). The addition of plant extracts had varying degrees of effect on the physicochemical quality of the yogurt. For example, increasing antioxidant properties, total phenolic content, acidity, and viscosity; however, the pH and syneresis of yogurt decreased compared to the control sample (117, 187, 188). According to Amirdivani and Baba (198), aqueous extracts of peppermint, dill, and basil probably enhanced the metabolic activity of yogurt bacteria, which improved milk’s fermentation and led to an increase in the acidity of yogurts. These fortified yogurts were characterized by a high content of bioactive peptides and improved antioxidant activities; additionally, in yogurt with peppermint extract, bacteria had the most increased proteolytic activity throughout fermentation and storage in the fridge (198). The antioxidant activity of yogurt is affected by bacterial fermentation, which results in the release of bioactive peptides (199).

According to Hasneen et al. (185), the lowest used concentration of turmeric, marjoram and sage extracts added to skimmed-milk yogurt, 1.0% (w/w), was the best overall organoleptically evaluated. The yogurt’s protein, fat, and ash contents fortified with the herbal extract showed no variation against control. Fortifying yogurt with 1.0% turmeric extract encouraged the growth of both bacterial yogurt strains (*Streptococcus thermophilus*, *Lactobacillus delbrueckii* ssp. *bulgaricus*). However, marjoram and sage extract slightly reduced their development than the control sample. There was a difference in titratable acidity; marjoram and sage extracts slowed acid growth in yogurt, resulting in higher pH values in the resultant products (185). Additionaly, the addition of rose extracts prolonged the time of preservation of the yogurt at 4°C, and the sensory and qualitative properties were maintained during 14-days refrigerating storage (182).

Srivastava et al. (200) prepared yogurts from different kinds of milk by varying the amounts of beetroot and ginger rhizome extracts. They discovered that the antioxidant activity increased with the concentration of the extracts (max 2%). In addition, the type of used milk can influence AA; the highest was detected in goats’ yogurts, followed by cow and buffalo milk yogurts (200). Tang et al. (189) used cinnamon leaf extract as a functional ingredient in stirred yogurt. Cinnamon extract’s bioactivity in yogurt was investigated, and there was a significant reduction in DPPH scavenging activity and ferric-reducing antioxidant power (FRAP) in yogurt. The antioxidant activity in yogurt was preserved from loss by encapsulating the extract (189).

Several researches added *Hibiscus sabdariffa* extract to dairy products since it has various bioactive components and high AA (181), improve the product’s sensory profiles, shelf life, and human health. According to Nuraeni et al. (178) addition of aqueous roselle extract to koumiss had no significant effect on the chemical or microbiological qualities of this fermented goat’s milk. Furthermore, roselle extract was used to prepare fortified goat-milk yogurt with anti-diabetic potency due to increased α-glucosidase inhibitory activity (183). It was also reported that roselle extract could suppress the growth of *Escherichia coli* and coliform bacteria *in vitro* and could be used in dairy products as an antibacterial agent and natural food colorant (178, 201). Additionally, roselle extract can be used to replace artificial red colorants in food because it contains water-soluble anthocyanins, which can be used as a natural color additive (202). Moreover, anthocyanins (E163) are approved as a natural colorant by the European Union. However, anthocyanins can be considered as more unstable molecules in the food product than synthetic dyes because their color stability is strongly influenced by various factors such as pH, high temperature, light, oxygen, solvents, enzymes, and other agents (203, 204). In addition, microencapsulation is an effective method for protecting the activity of this group of phenolic compounds (205). According to Rozan et al. (181), combining hibiscus extract with carrot juice resulted in a functional yogurt with improved color and extended shelf life of 21 days. In addition, developed yogurt had increased total phenolic compounds and total carotenoids (181).

Rosemary extract (E392) might be used in different dairy products, such as UHT milk, dehydrated milk, flavored fermented milk products, creams, unripened cheese, and processed cheese (206). Commercial items with rosemary extract are cheese (Fabriqué par Fromagerie Neufchâteau, France), halloumi cheese (Dodoni, United Kingdom), cheese (St Môret, France), Apérifrais (Tartare, Switzerland), French Roulé (Chêne d’argent, France), fresh creamy cheese (Rians, United States), whipped Philadelphia (Philadelphia, France), buffalo cheese (Milton G. Waldbaum Company, United States), and creamy gorgonzola dip (Finesse Records, United States). Another food additive is beetroot red (E162), which is used as a coloring in various dairy products; for example, milblu creamy yogurt (Kaufland, Germany), strawberry milk (Dairy Manor, United Kingdom), strawberry yogurt (Yoplait, France), frozen dessert (Ski, France), strawberry yogurt drinks (Nestle, United Kingdom), and raspberry protein snack (Valio, Spain). In addition, fermented milk with the addition of aqueous lemongrass extract (Activia, Danone) is currently available on the market.

### 2.5. Beverages

The fortification of beverages is another use for plant extracts. Pluháčková et al. (2020) analyzed beers that were enriched with five different herbal extracts and were evaluated as potentially benefitial to consumers’ health (207). Table 6 presents the name of plants and herbs, basic information about the preparation of extracts, and concentrations of them applied in beverages. The addition of medicinal plant extracts to beer increased the total content of polyphenols, some phenolic acids, and essential oils. Camphor and thujone were the most abundant essential oils in beer fortified with sage extract, whereas beer with chamomile extract included α-bisabolol, p-cymene, limonene, and farnesene. Even a low concentration of herbal extracts impacted fortified beer’s scent, flavor, and overall perception (207).

**Table 6:** Application of plant extracts in beverages.

Plant	Extract	Application	Concentration	Reference
*Hibiscus sabdarifa* L.	Aqueous extract: dried and grounded roselle calyces and water in a ratio of 1:10, extraction at 50°C for 30 min, filtration	Formulation of roselle extract-fruit juice blends (guava, papaya, and mango); low-cost, functional beverage with improved antioxidant properties and mineral composition	20%, 40%, 60%, 80%	(208)
*Ocimum sanctum* L.	Aqueous extract: 10–15 g of fresh or dried plants (leaves, root) were added to 200 mL of water, then boiled and filtered	Orange wine fortification with herbal extracts; to enhance its sensory properties and health benefits for consumers	35 mL extract to 200 mL of wine	(209)
*Cymbopogon citratus*			30 mL extract to 200 mL of wine	
*Mentha arvensis* L.			25 mL extract to 200 mL of wine	
*Zingiber officinale* (root)			20 mL extract to 200 mL of wine	
*Mentha piperita* L. *Melissa officinalis* L. *Origanum vulgare* L. *Thymus serpillum* L. *Salvia officinalis* L.	Aqueous extract: 1 g of dried herbal powder in 100 mL water for 5 min, filtration	Apple juice enrichment with herbal extract, measurement of antioxidant activity, total phenolics and flavonoids, and sensory analysis	40%	(210)
*Lippia citriodora Ilex paraguarensis Eugenia caryophyllata Camellia sinensis*	Herb with water 1% (w/v) was brewed for 5 min at 98°C	Lemonade with herbal extracts that are high in TPC and ascorbic acid; improved stability, sensory, and nutritional characteristics	5%	(211)
*Tilia argentea Zingiber officinale Mentha piperita Erica arborea*			10%	
*Phyllanthus niruri*	5 g of fresh crushed leaves were boiled with 100 mL of water and then filtered	Development of a functional amLa wine with improved nutritional and health benefits with sensory qualities similar to commercial wine	5, 10, 15 and 20 mL extract	(212)
*Olea europaea* L.	Infusion: 75 g/L of leaves, powder (atomized extract): 10 g of powder made from 1 l of infusion (33 g/L of leaves)	The effect of olive leaf extracts on the bitterness, AA, and colloidal stability of beer; pleasant sensory profile (flavor and aroma)	Infusion: 0.34 and 1.01 g/L, or 132 mL/L (corresponding to 9.9 g/L leaves); powder: 0.34, 1.01 or 3 g/L	(213)
*Plantago lanceolata* L. (leaves)	Ethanolic extract: maceration in 60% (v/v) ethanol solution for 30 days in the dark	Determination of active components (phenolics and essential oils) and sensory acceptability of herbal-fortified beer	10 mL extract to 1 L of beer	(207)
*Tilia cordata* Mill. (flowers)			40 mL extract to 1 L of beer	
*Echinacea purpurea* L. (root) *Matricaria Chamomilla* L. (flowers) *Salvia officinalis* L. (leaves)			14 mL extract to 1 l of beer	

Guglielmotti et al. (2020) prepared beers with infusion or atomized extract of olive leaves, which increased the total polyphenol content of the beers. However, high polyphenol concentration might negatively affect beer’s colloidal stability and quality. Nonbiological haze development may arise during storage because of protein-polyphenol interactions (213).

Soaking herbs in distillates or wine is a popular procedure across the world, producing a variety of extracts known as herbal liquors and wines (214, 215). Wine is a functional fermented beverage that might be fortified with herbal extracts to attain health benefits. For example, Purkait and Pandey (216) prepared enriched red wine with 10% roselle and 6% peppermint extract which panelists preferred above wine without herbs. In addition, adding dried roselle into this fermented beverage increases its acidity, which may improve microbial spoiling resistance and storability. Furthermore, plant extracts can also be added to fruit wines created with a non-grape juice base. For example, the addition of four extracts (lemongrass, tulsi, ginger, and peppermint) into orange wine has resulted in a new product with improved quality, increased acceptability, and broader applications. These herbal extracts enhance the basic characteristics of citrus wine when used in specific amounts (209). Another health-functional fruit wine was amLa wine with 15 mL of *Phyllanthus niruri* extract; that beverage recorded the highest overall acceptability due to its perfect combination of taste, fragrance, and appearance (212). The highest concentration of ascorbic acid, tannins, and phenolics was observed in amLa wine with 20 mL of herbal extract; furthermore, this wine had the most increased free radical scavenging activity (212).

The health benefits of fruit juices have been related to their high level of antioxidant molecules; hence, several studies suggest that the high content of added antioxidant sources has been linked to the beneficial properties of fruit juices (217). Plant extracts can also be applied to fruit juices to increase and supplement the health benefits of fruits (218, 219). For example, apple juice with mint extract rich in bioactive molecules showed antioxidant activity; moreover, the panelists rated this enriched juice higher in pleasing smell and taste than pure juice (210). Additionally, Mgaya Kilima et al. (208) reported that combining tropical fruit juices with roselle extract has improved the mineral composition, total monomeric anthocyanins, and total phenol content in the blends based on the concentration of extracts added to the final products. On the other hand, vitamin C content in blends was lower than in individual fruit juices, and a similar trend was also described by Fasoyiro et al. (220). Tamer et al. (211) prepared lemonade with linden, ginger, peppermint, or heather extract. The addition of these plant extracts increased the product’s acceptability, the content of phenolics, and the antioxidant activity. Adding herbal extracts with a high antioxidant capacity to lemonades can contribute to the stability of juice against oxidation but also affect its color (217). Natural antioxidants, such as ascorbic acid, might be employed to improve the color fastness of fruit juices and the stability of carotenoids (221, 222).

Plant extracts are used in beverages to add fragrance, color, flavor, and frothiness. Consumers might be attracted to the extract’s unique natural flavor, which enhances the taste of a drink. For example, cranberry extract powder was initially applicated in different types of beverages and by a variety of population age categories; however, since 2017, the EFSA has allowed its use only for adults and excluded all children, from infants to adolescents (98, 223). Furthermore, anthocyanins might be used as a natural coloring agent, under the additive-number E163, in acidic beverages such as fruit or aromatized wines, cider, flavored and carbonated soft drinks (224, 225). In addition, this natural coloring additive is available in various forms like E163a—cyanidin (red), E163b—delphinidin (blue), E163c—malvidin (purple), E164d—elargonidin (orange), E164e—peonidin (red-brown), and E165f—petunidin (dark red) (225). Another food additive is an aqueous extract of quillaia (E999), which is used as the foaming agent in non-alcoholic flavored beverages, beers, cider, and perry; in addition, the maximum concentration in these beverages is 200 mg/L (226). Examples of commercial applications of this food additive are ginger beer (Ceylon Cold Stores PLC, Sri Lanka), root beer (A&W, United States), cream soda (Crush, Canada), and Nitro Pepsi (Pepsi, United States). Furthermore, the peach passion drink (Tim Hortons, Canada) contained quillaia and paprika extracts.

Rosemary extract (E392) was used in different beverages such as orangina schweppes (Schweppes, France), non-alcoholic beer (Tourtel, France), protein powder (Orgain, Germany), pomegranate lemonade (Turkey Hill, United States), cocktail mixer (Hella cocktail co, United States), and kombucha (Kevita Inc., United States). Moreover, Life aid drink (LIFEAID Beverage Co., United States) contains seven different extracts obtained from turmeric, rosemary, ginger, oregano, stevia, piperine, and cayenne pepper. The limitation of the use of rosemary extract in beverages are, e.g., table waters and flavored sports drinks (MPL 500 mg/L); flavored drinks (MPL 700 mg/L); cider and perry, mead, spirit beverages, fruit wine, aromatized wines, and alcoholic drinks with less than 15% of ethanol (MPL 1,000 mg/L); chocolate and malt dairy-based drinks, coffee-based drinks, and instant tea (MPL 2,000 mg/L); whey protein—sports drinks (MPL 4,000 mg/L); and vegetable protein drinks (MPL 20,000 mg/kg) (206).

Other drinks where plant extracts have been applied are still water with herbal extract (Rajec, Czech Republic), drink with chrysanthemum flower and Pu′er tea leaves extracts (Yeo’s, Malaysia), an energy drink with guarana extract (Big Shock s.r.o., Czech Republic), and a milk tea drink (MineShine, Taiwan), which contains black tea (leaves) and barley extracts. Moreover, alcoholic beverages also contain different plant extracts; for example, sparkling wine with natural lychee extract (Café de Paris, France), white wine with rose macerate (Rybízák s.r.o., Czech Republic), vermouth with extract of aromatic herbs (Martini, Italy), sangria with citrus extract (Dom Josué, France), and cider with lavender extract (Joker Cider s.r.o, Czech Republic).

## 3. Discussion

Consumers’ dietary preferences are changing, and more consumers prefer naturally processed and safe products without synthetic food additives. In addition, plant extracts are becoming more popular in the food industry because of their low-cost, functional characteristics, and in some cases, renewable source of biologically active components (227). Moreover, extracts have a concentrated taste and flavor and a longer shelf life than fresh plants, making them ideal as ingredients for food products. Natural antioxidants found in these extracts have the potential to fortify food, and extend its shelf life, improve nutritional and sensory properties. Herbal extracts could inhibit or slow the oxidation of lipids, but they can also affect the qualitative properties of food products in both positive and negative ways. As a result, only relevant toxicologically tested extracts should be used to develop novel fortified products, followed by sensory analysis. The human senses are used to evaluate product attributes, and consumer perceptions and reactions to product attributes are studied. Some herbal extracts can give a distinctive food taste that may be unacceptable to consumers; hence, sensory quality is one of the important aspects of successful marketing (86, 228). From a commercial perspective, the overall acceptability of a product is often more important than evaluating the enriched product in terms of antimicrobial activity and slowing or inhibiting lipid oxidation. Additionally, the traditional application of natural extract directly on the food surface, such as spraying, pulverization, and dipping, might be replaced by incorporating extract and its active antimicrobial compounds into active packaging material. These novel active packaging could help extend food shelf life and prevent changes in taste caused by excessive amounts of bioactive compounds (11, 156, 229, 230). This allows the packaging film to become a dynamic and interactive element of the preservation procedure of food products (231).

As shown in the previous tables, several researchers extracted many beneficial active compounds from food waste, and then they applied extracts to new products (135, 140, 153, 184). There is currently a global trend of utilizing co-product or waste, which arose in a considerable amount during food production, and extracting functional compounds from it can help reduce the environmental burden of waste while also adding value to the developed functional food product (232).

According to Lourenç et al. (92), plant extracts that inhibit oxidation should also be non-toxic and suitable for human consumption; furthermore, the application of plant extracts should be effective already at low concentrations, between 0.001 and 0.01%, because organoleptic properties such as flavor, odor, color, and stability of the food matrix will probably not be affected negatively. Finally, the extract’s economic aspect and stability during its processing, storage, and as part of food are also important. The stability of polyphenols is affected by the pH value; for example, an aqueous solution with tea catechins was more stable at a highly acidic pH below 4 than at a slightly acidic higher than pH 6 (233). Furthermore, acid pretreatment may improve anthocyanins’ stability by creating a more favorable extraction environment and inactivating enzymes capable of destroying anthocyanins (234). In an acidic aqueous environment, anthocyanins exist in equilibrium, while their degradation occurs as the pH increases above 6 (233).

The supercritical fluid extraction (SFE) approach is utilized on an industrial scale, particularly for essential oil extraction; however, following a potential modification, this technique might be used to extract non-polar compounds (235). SFE is an efficient method that might be used for extracting phenolic molecules and flavonoids (69).

Bioactive compounds such as flavonoids, phenolic acids, essential oils, tannins, carotenoids, organosulfur compounds, phytosterols, and tocopherols are used in the food industry in the processing of vegetable oils, meat and seafood products, pastries, dairy products, beverages, etc. (236). Additionally, plant extracts may contain up to thousands of different molecules and determine the constituents responsible for a specific biological effect can be challenging since mixtures’ behavior cannot always be characterized by the presence of only a few identified compounds (237). In addition, herbal extract as a whole or several herbs in complex formulations could show better efficacies than equivalent doses of individual bioactive molecules or single herb (238).

It is important to remember that medical herbs and their mixtures can pose a health risk due to non-essential and toxic metals, such as lead, cadmium, aluminum, mercury, or chromium, which can accumulate in plants (239). These toxic metals can be extracted into the infusion during its preparation and can be hazardous to consumers’ health. The maximum values for these metals in food supplements are defined by Commission Regulation (EC) No 629/2008, as amended, notably lead (3.0 mg·kg^−1^), cadmium (1.0 mg·kg^−1^), and mercury (0.10 mg·kg^−1^) (240). Their overall concentration mostly determines the release of metals into the herb infusion, the metal’s affinity for the herb matrix, the metal’s solubility in solution, and the extractability of the components at high temperatures (77, 241). Some toxic metals, such as arsenic and lead, could be organically bound by biomacromolecules - polyphenols, with which they form ligands, and therefore are not biologically available in plant infusions. As a result, infusions could be less hazardous (241, 242). For example, Arpadjan et al. (242) observed that lead might not enter the infusions made from lead-contaminated plants since it is often weakly soluble in water and has a very low extraction factor. On the other hand, ethyl alcohol as a solvent showed a higher extraction power than water because ethanol could dissociate organic matrices and release bounded lead (243).

The techniques used to produce fortified food should be developed to avoid protein denaturation or nutritional loss. Furthermore, it is necessary to choose the appropriate application of plant extracts to food because a good approach can improve the efficiency of the extracts even at a lower dose. For example, lyophilization or extract encapsulation techniques appear acceptable for preparing extract to fortify food. Freeze drying was one of the most popular methods for preserving bioactive compounds in plant extracts used as food ingredients in analyzed studies.

A food product fortified with microencapsulated extract may have a better sensory evaluation than a free extract due to masking the aroma and taste of plant material (117, 244). Furthermore, the encapsulation method might prevent compounds from being vulnerable to the digestive process and the AA of the extract from being lost during digestion (189). The extract’s encapsulation can potentially preserve its bioactive properties and increase its shelf life, storage stability, bioavailability, and solubility, as well as reduce hygroscopicity and retention of microstructure (245). However, the size of the prepared particles can affect the acceptability of the novel food product fortified with them, as larger particles can cause a gritty-mouth feeling (246).

The stability of polyphenols in extracts is crucial for various food applications because they might be easily influenced by oxygen, temperature, pH, light, enzymes, and other factors (247). Polyphenols are recognized as beneficial phytochemicals for human health; nevertheless, these compounds can interact with proteins, carbohydrates, lipids, and other food matrix components, which might significantly affect the nutritional and nutraceutical potential of fortified products (248–251). For example, tannins might develop tannin–protein complexes, which reduces their antioxidant ability (44, 45). Because of the complexity of polyphenol interactions within the food matrix (252), it is important that each food matrix be assessed individually regarding its interacting phenolic compounds throughout the development of functional food enriched with phenolic-rich plant extract. Interactions of bioactive compounds with other food ingredients could limit their full release during gastrointestinal digestion. Moreover, the polyphenols’ bioavailability is a limiting factor of their efficacy in preventing some diseases because these molecules’ chemical structure and biological activity might be transformed during metabolic processes and interaction with colon microbiota (247, 253).

Furthermore, there are countless studies regarding antioxidants of natural origin in the literature; nevertheless, they have not been completely investigated their toxicity and the amount of biologically active compounds reaching the targeted spot in the human body. Future use of plant extracts as antimicrobial or antioxidant agents to preserve food requires that their efficacy as a food additive be proven and regulatory approved. Food fortified with plant extracts might offer health benefits; in that case, further research on *in vitro* tests (e.g., simultaneous digestion) and animal trials is needed to assess the health advantages to consumers.

We can find the use of extracts in botanical ingredients, such as plant extract in food and beverages, is growing worldwide. Currently, the most popular applications of plant extract are mainly non-alcoholic and alcoholic beverages. We can also find confectionery, pastries, and dairy products with botanicals, such as cheese and yogurt.

According to KerryDigest’s study, just 11% of European customers believe they completely understand all the benefits of botanicals; consequently, they recommend further education regarding botanical ingredients and how they may enhance human health (254). According to Future Market Insight reports, the global food market is expected to grow from USD 10.9 billion in 2022 to USD 34.5 billion in 2032, exhibiting a compound annual growth rate (CAGR) of 12.2% (255). More specifically, the global market for food botanicals demand is estimated to grow at 3.2% CAGR in these 10 years. The food botanicals market is expected to surpass USD 1.95 billion by the end of 2032. In conclusion, even though the previously mentioned study included just oleoresins, essential oils, and dried herbs from various sources (plants, algae, fungi, or lichens), we anticipate market growth in food and beverages with applied plant extract (256). Moreover, López-Rodríguez et al. (2022) assume that plant-origin extracts will be the most likely examined group in the scientific literature because this type of extracts has been the most approved as novel food in the last 5 years more than algal, fungal, and animal-origin extracts (98).

## 4. Conclusion

In conclusion, the use of plant extracts in the food industry has received significant interest in recent years. Plant extracts consist of functional and sensory properties such as nutraceutical, antioxidant (radical scavenger) and antimicrobial activities, and attractive color and flavor, making them suitable for use as a natural additive for food applications. They are also used in food applications since they are non-toxic, affordable, readily available, and environmentally acceptable. Plant extracts with high AA may be commercialized and applied in various food formulations because they might improve the product’s qualities and make it functional food. Moreover, these extracts might be options for replacing synthetic compounds, which could be potentially harmful to human health. On the other hand, it is important to consider that plant extracts used as food ingredients may present various challenges related to their stability, purity levels, compatibility with food matrix, regulatory limitations, and cost-based aspects (extraction and application should be economical).

## Author contributions

AP: conceptualization, software, writing—original draft preparation, and visualization. JM: writing—review and editing, supervision, project administration, and funding acquisition. All authors contributed to the article and approved the submitted version.

## Funding

This research was supported by the internal grant of Tomas Bata University in Zlin, grant number IGA/FT/2023/003.

## Conflict of interest

The authors declare that the research was conducted in the absence of any commercial or financial relationships that could be construed as a potential conflict of interest.

## Publisher’s note

All claims expressed in this article are solely those of the authors and do not necessarily represent those of their affiliated organizations, or those of the publisher, the editors and the reviewers. Any product that may be evaluated in this article, or claim that may be made by its manufacturer, is not guaranteed or endorsed by the publisher.
